# Challenges in autoimmune polyendocrine syndrome type 2 with the full triad induced by anti-programmed cell death 1: a case report and review of the literature

**DOI:** 10.3389/fimmu.2024.1366335

**Published:** 2024-04-18

**Authors:** Qin Pan, Ping Li

**Affiliations:** ^1^ Department of Endocrinology, Chengdu Eighth People’s Hospital (Geriatric Hospital of Chengdu Medical College), Chengdu, Sichuan, China; ^2^ Department of Endocrinology, Shengjing Hospital of China Medical University, Shenyang, Liaoning, China

**Keywords:** anti-programmed cell death protein 1, autoimmune polyendocrine syndrome type 2, adrenal insufficiency, adrenal crisis, hypothyroidism, type 1 diabetes mellitus

## Abstract

**Background:**

Immune checkpoint inhibitors (ICPis) induce autoimmune diseases, including autoimmune polyendocrine syndrome type 2 (APS-2), which is defined as a combination of at least two of the following endocrinopathies: autoimmune thyroid disease, type 1 diabetes, and Addison’s disease. Cases with the full triad are rare. We present a case of an elderly woman who developed APS-2 with the complete triad shortly after starting anti-programmed cell death 1 (anti-PD1) treatment and review the related literature.

**Case:**

A 60-year-old woman, without any personal or family history of autoimmune and endocrine diseases, started the immunotherapy of anti-PD1 (camrelizumab) for squamous cell carcinoma of the urethral meatus. She developed primary hypothyroidism with elevated antibodies to thyroid peroxidase and thyroglobulin after 25 weeks of treatment, and developed primary adrenal insufficiency with adrenal crisis and fulminant type 1 diabetes with ketoacidosis after 45 weeks. Therefore, this patient met the diagnosis of APS-2 and was given multiple hormone replacement including glucocorticoid, levothyroxine and insulin therapy. Continuous improvement was achieved through regular monitoring and titration of the dosage.

**Conclusions:**

Different components of APS-2 may appear at different time points after anti-PD1 administration, and can be acute and life-threatening. A good prognosis can be obtained by appropriate replacement with multiple hormones.

**Insights:**

With the clinical application of ICPis to APS-2, the complexity of its treatment should be paid enough attention.

## Introduction

Immune checkpoint inhibitors (ICPis) are novel antitumor agents. Over-activated immune cells may also lead to clinical manifestations of autoimmune abnormalities in multiple systems of the body, namely immune-related adverse events (irAEs) (1). Endocrine glands are common targets of irAEs, particularly the thyroid and pituitary. A meta-analysis has shown that patients treated with anti-programmed cell death protein 1 (c) combined with anti-cytotoxic T-lymphocyte antigen 4 (anti-CTLA-4) had the highest incidence of hypothyroidism and hyperthyroidism, patients treated with anti-PD1 alone were at even higher risk of hypothyroidism, and patients treated with anti-CTLA-4 alone were at even higher risk of developing pituitary inflammation (2). With the gradual increase in clinical use of ICPis, reports on the involvement of two or more glands are becoming more common. ICPis therapy can usually be continued with close monitoring. However, moderate to severe irAEs may cause severe organ dysfunction, a reduced quality of life, and even death, requiring early recognition and appropriate management (3).

Here, we present a case of autoimmune polyendocrine syndrome type 2 (APS-2) after anti-PD1 treatment in a patient with squamous carcinoma of the urethral meatus. APS-2 is defined as the occurrence of any type 1 diabetes mellitus (T1DM), autoimmune thyroiditis, and primary adrenal insufficiency (Addison’s disease). In this case, the patient presented with primary hypothyroidism and T1DM with ketoacidosis and primary adrenal insufficiency with adrenal crisis, i.e., all components of APS-2 were present. In this paper, we will present the clinical manifestations and treatment of this special case and also review the reported cases of anti-PD1-induced APS-2 in the literature, to help clinicians improve their awareness and understanding of this disease when anti-PD1 is widely used today.

## Case description

1. The baseline situation: A 60-year-old female, without any personal or family history of autoimmune and endocrine diseases, was diagnosed with squamous cell carcinoma of the urethral meatus with inguinal lymph node metastasis and was treated with camrelizumab (anti-PD1, 200mg every 3 weeks) 14 times combined with anlotinib hydrochloride capsules (12mg/day) at regular intervals from February to December 2020. Before anti-PD1 administration, blood glucose level and thyroid function was normal except for a mild increase in thyroglobulin antibody (TgAb). However, the baseline adrenal function and islet function were not examined. The first application of anti-PD1 was on February 12, 2020. After the initial use of anti-PD1, the oncologist regularly monitored the patient’s thyroid function, adrenal function and blood glucose and found them to be within the normal range.

2. The progress of APS-2:

(1) The first occurrence of hypothyroidism: At the 25^th^ week after anti-PD1 administration, a significant increase in thyroid autoimmune antibodies (ARCHITECT Anti-Tg/Anti-TPO Reagent Kit, Chemiluminescent Micro-Particle Immunoassay) combined with hypothyroidism was found, while adrenal function remained normal during the same period, and growth hormone (GH) and sex hormone were not tested. The patient started on levothyroxine sodium tablets (L-T4) 12.5ug/d and gradually increased to 75ug/d., When tested one month after taking L-T4, thyroid function returned to normal, so 75ug/d was continued.

(2) The simultaneous occurrence of T1DM and Addison’s disease: At the 45^th^ week after anti-PD1 administration, the patient developed fatigue, nausea, epigastric discomfort, and vomiting, accompanied by weight loss (5 kg in 1 week). Physical examination was as follows: height 165 cm, weight 45 kg, body mass index 16.5 kg/m, body temperature 36.3°C, pulse 120 beats/min, respiration 30 breaths/min, blood pressure 127/92 mmHg, Although mentally lethargic, the patient can answer the doctor’s question correctly. No alopecia, pigmentation, vitiligo, oedema or bleeding spots on the skin and mucous membranes. No abnormalities on thyroid inspection and palpation. Heart rate 120 beats/min with normal rhythm. Mild tenderness appeared in the upper abdomen without rebound pain or muscle tension. No muscle weakness or other nervous system abnormalities were found in physical examinations.

**Figure 1:**
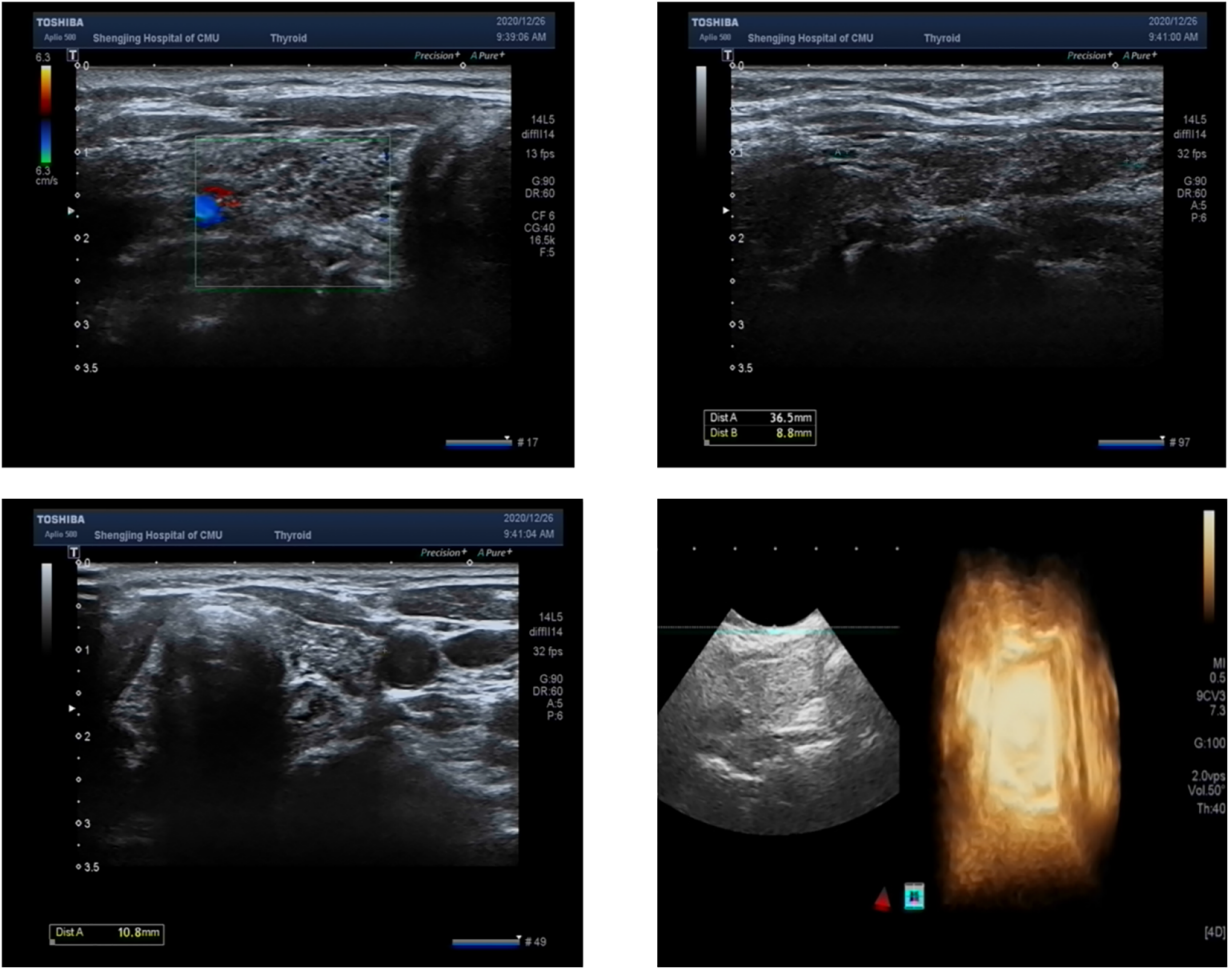
Ultrasound showing normal thyroid and parathyroid glands.

**Figure 2:**
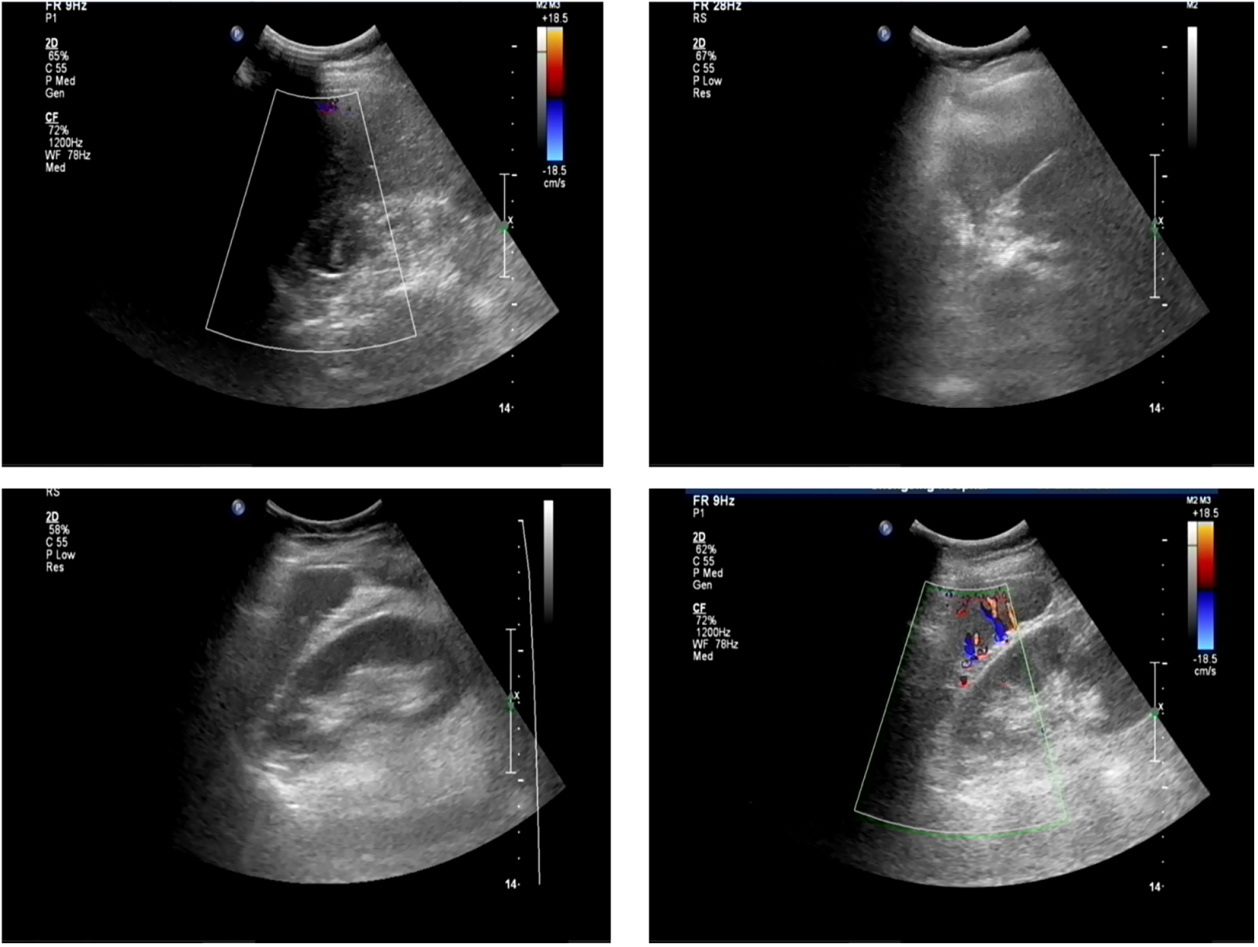
Ultrasound showing normal bilateral adrenal glands.

**Figure 3:**
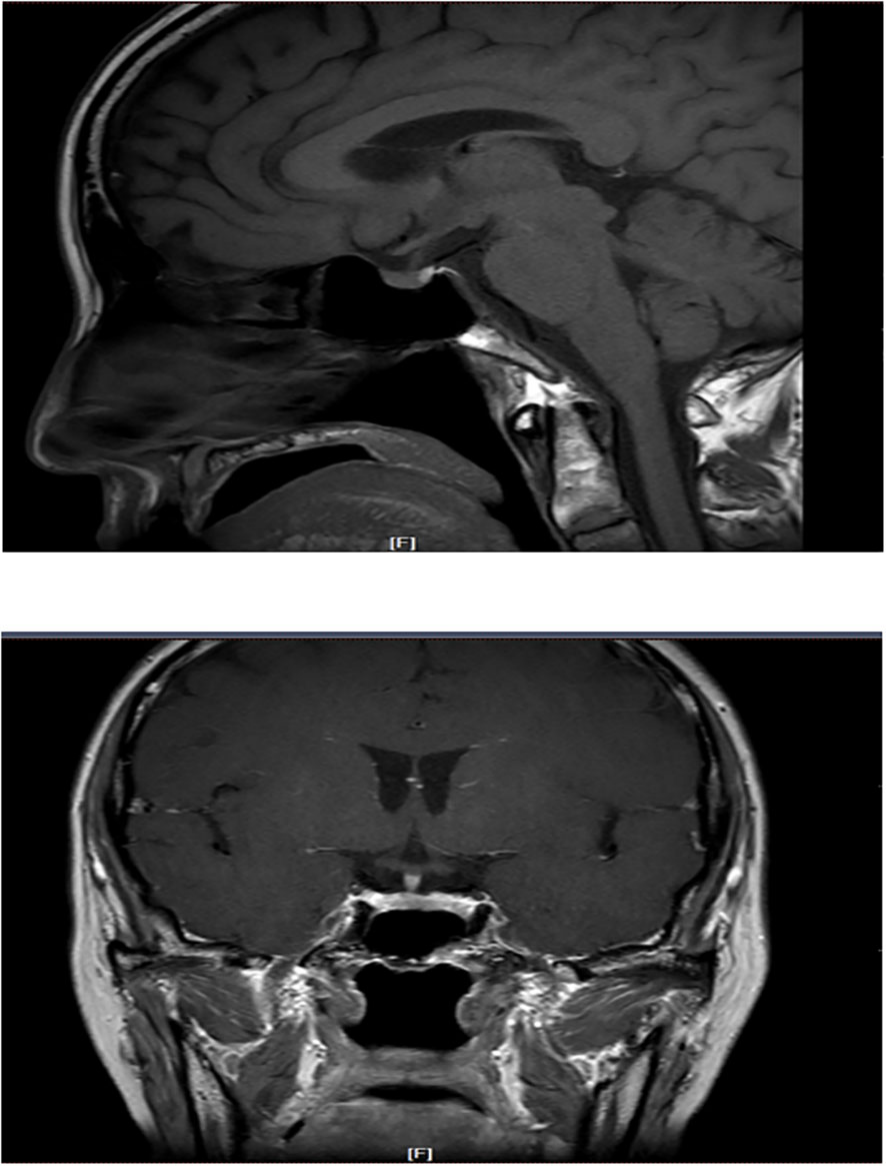
MRI with contrast showing a normal-sized pituitary and normal enhancement.

The patient had significantly elevated blood glucose, with fasting glucose of 21.06 mmol/L, reduced fasting C-peptide and insulin, urinary ketone bodies 4+, and significantly lower PH indicating diabetic ketoacidosis (DKA), and then admitted to the hospital. Other laboratory and imaging findings included negative autoimmune antibodies for type 1 diabetes (Line Immuno Assay for the Detection of Antibodies in Autoimmune Diabetes, Western blotting), normal levels of sex hormone, human growth hormone (GH), insulin growth factor-1 (IGF-1), parathyroid hormone (PTH), serum transaminase and creatinine (**Table 1**). The ultrasound indicated that the echo of thyroid tissue was rough without other obvious abnormality in the thyroid or parathyroid area (**Figure 1**). Also, the ultrasound indicated no obvious lesions in bilateral adrenals (**Figure 2**). Contrast-enhanced magnetic resonance imaging (MRI) of pituitary indicated no definite abnormalities (**Figure 3**).

**Table 1:** Laboratory and hormonal test results.

Test	Baseline ^a^	Week 12	Onset of primary hypothyroidism(week 25)	Onset of TIDM and Addison’s disease (week 45)	Week 79	Reference Range
FT3 (pmol/L)^b^	5.36↑	15.49↑	<1.64↓	5.79↑	2.87	2.63–5.71
FT4 (pmol/L)^b^	14.82	4.96↓	5.27↓	15.5	14.24	9.01–19.05
TSH (uIU/mL)^b^	2.23	0.8792	>100↑	1.3773	15.6720↑	0.3–4.8
TPOAb (IU/mL)	5.30	12.67↑	412.65↑	174.24↑	8.60↑	0–5.61
TgAb (IU/mL)	149.45↑	>1000.00↑	>1000.00↑	>1000.00↑	37.92↑	0–4.11
Cortisol (µg/dL)	–	14.88	–	3.41↓	9.00	6.02–18.4
ACTH (pg/mL)	–	41.91	–	313.16↑	10.59	7.2–63.3
Estradiol(pg/mL)	–	–	–	<15.0	–	–
Testosterone (ng/mL)	–	–	–	0.21	–	–
LH (mIU/mL)	–	–	–	54.70	–	10.87–58.64
FSH (mIU/mL)	–	–	–	96.83	–	16.74–113.59
PRL (ng/mL)	–	–	–	7.27	–	2.74–19.64
Prog (ng/mL)	–	–	–	0.29	–	0.01–0.78
GH (ng/mL)	–	–	–	1.274	–	0.014–5.219
IGF-1 (ng/mL)	–	–	–	43.29	–	60–350
PTH(pg/mL)	–	–	–	27.0	–	12–88
FBG^b^	–	6.1 mmol/L (109.8 mg/dL)	–	25.72 mmol/L↑ (462.96 mg/dL)	–	3.9-6.1 mmol/L (70.2–109.98 mg/dL)
FCP (ng/mL)	–	–	–	0.15	–	0.78–5.19
Fins (mU/L)	–	–	–	0.3	–	2.3–11.8
GA (%) ^b^	–	–	–	20.05	–	–
HbA1c (%)^b^	–	–	–	5.7	7.6	–
K+ (mmol/L)^b^	3.9	2.93	–	5.66	3.71	3.5–5.5
Na+ (mmol/L)^b^	141.5	139.7↑	–	126	128.3	136–145
Cl−(mmol/L)^b^	103.0	103.9	–	92.6	94.1	96–108
GADA	–	–	–	negative	–	–
IA2A	–	–	–	negative	–	–
ICA	–	–	–	negative	–	–
IAA	–	–	–	negative	–	–
Amylase				145.6	47.4	35–135
Lipase	–	–	–	269.7	8.6	5.6–51.3

Baseline ^a^ referred to the time before the initial use of anti-PD1. ^b^, the measurement of electrolytes, FPG, GA, HbA1c and thyroid function were much more frequent than we can show, and we selected the values of the above several time points for display in this table. “-” means that the value was not measured. Abbreviations: FT4, free thyroxine; FT3, free triiodothyroxine; TSH, thyroid stimulating hormone; TPOAb, thyroid peroxidase antibody; TgAb, thyroglobulin antibody; ACTH, adrenocorticotrophic hormone; LH, luteinizing hormone; FSH, follicle-stimulating hormone; PRL, prolactin; Prog, progesterone; GH, human growth hormone; IGF-1, insulin-like growth factor 1; PTH, parathyroid hormone; FBG, fasting blood glucose; FCP, fasting c-peptide; Fins. fasting insulin; HbA1c, glycated haemoglobin; GADA, glutamic acid decarboxylase antibody; IA2A, insulinoma-associated protein 2 antibody; ICA, islet cell antibody; IAA, insulin antibody; DKA, adrenal cortex antibody. "↑" means higher than the reference range and "↓" means lower than the reference range.

(3) Diagnosis: Considering the patient had no previous history of diabetes, the sudden onset of T1DM and DKA were speculated to be drug-induced. The patient had an acute onset of DKA, fasting blood glucose > 16mmol/L, HbA1c < 8.5% and serum fasting C-peptide < 0.3ng/mL, which met the diagnostic criteria of fulminant type 1 diabetes. Decreased blood cortisol and significantly increased ACTH was found accompanied by low sodium, which supported the appearance of primary adrenal insufficiency (Addison’s disease). Considering the patient’s concurrent severe symptoms of nausea, vomiting, and lethargy, we diagnosed adrenal crisis. The patient had T1DM, primary hypothyroidism together with Addison’s disease, therefore, she can be diagnosed as APS-2 secondary to anti PD-1 administration.

(4) Treatment: The treatment of DKA included sufficient liquid supplementation, insulin therapy, and immediate correction of ionic disturbances. Finally, due to economic reasons, the patient did not choose insulin pump, but chose subcutaneous injection of short-acting insulin three times a day and long-acting insulin once a day for insulin replacement. The treatment of adrenal crisis involved sufficient liquid supplementation, glucocorticoid replacement including hydrocortisone intravenous infusion 200 mg/day and gradual reduction to oral dosage of 15mg/day. After pituitary crisis and ketoacidosis were corrected, the patient’s condition was greatly improved and allowed to leave the hospital.

3. Follow-up: This patient was given multiple hormone replacement including: (1) glucocorticoid replacement: 10 mg hydrocortisone p.o. in the morning and 5 mg p.o. in the afternoon, and was suggested to increase the glucocorticoid dose by 3–5 times when under stress; (2) thyroid hormone supplementation: levothyroxine 75 µg p.o. in the morning; (3) insulin therapy of T1DM: insulin glargine 7 U subcutaneously before bedtime, insulin aspart 4 U before three meals, and the patient was taught and trained to titrate insulin dosage according to blood sugar levels. The patient’s blood glucose fluctuated between 4 and 14 mmol/L (72-252mg/dL), with occasional appearance of hypoglycaemia. About six 6 months after discharge from the hospital, the patient measured HbA1c 7.6%, and the thyroid function and serum ion levels were in the normal range. Up to the last visit in our hospital, the hormone supplements remained the same dose.

As to the treatment of squamous cell carcinoma of the urethral meatus, the patient refused to continue using anti-PD1 because of the occurrence of APS-2. After full discussion with the doctor, the patient still chose to start chemotherapy (cisplatin combined with gemcitabine hydrochloride, which was adjusted to gemcitabine monotherapy due to poor tolerance). Six months later, local boost radiotherapy was performed, and the tumor was reduced after radiotherapy. Eleven months after discharge, the tumor progressed and the physical condition was not good. The patient was given Paclitaxel liposome for injection chemotherapy until January 27, 2022, when the last chemotherapy was performed. Since the last visit, the patient could not be contacted and was lost to follow-up.

## Discussion and conclusions

This is a case of APS-2 with the complete triad (type 1 diabetes, hypothyroidism, and adrenal insufficiency) following treatment with anti-PD1. In the first reported case (4), a patient in their 70s developed the full triad of APS-2 shortly after commencing anti-PD1 for unresectable melanoma in 2019. The primary manifestation of APS is the impaired function of multiple endocrine glands caused by autoimmune reactions. APS-2 is more frequent in women and usually develops in the 3rd to 4th decade of life. It is thought to be polygenic and is associated with mutations in cytotoxic T lymphocyte-associated antigen 4 (CTLA-4) and human leukocyte antigen (HLA) DR3 and DR4 serotypes (5).

The initial endocrine gland injury in this patient involved the thyroid. The patient developed hypothyroidism 25 weeks after the first use of camrelizumab, presenting as significantly elevated TSH, TPOAb, and TgAb titres. The patient was diagnosed with primary hypothyroidism secondary to autoimmune destruction of the thyroid due to pharmacological factors rather than damage to the anterior pituitary. Six months after the onset of primary hypothyroidism, the patient developed fulminant type 1 diabetes with diabetic ketoacidosis as the first symptom. The patient met all three diagnostic criteria established in a 2007 Japanese study of fulminant type 1 diabetes (6): (1) Occurrence of diabetic ketosis or ketoacidosis soon (around 7 days) after the onset of hyperglycaemic symptoms (elevation of urinary and/or serum ketone bodies at first visit); (2)Plasma glucose level ≥16.0 mmol/l (>288 mg/dl) and glycated haemoglobin (HbA1c) level <8.5% at first visit; (3) Urinary C-peptide excretion <10 μg/day or fasting serum C-peptide level <0.3 ng/ml (<0.10 nmol/l) and <0.5 ng/ml (<0.17 nmol/l) after intravenous glucagon (or after meal) load at onset. Under the stress of diabetic ketoacidosis, the patient exhibited a potential latent adrenocortical insufficiency with an initial presentation of “adrenal crisis.” We need to distinguish whether this adrenal insufficiency is primary or secondary. Common laboratory findings of primary adrenal insufficiency are a random or early-morning decrease in cortisol and an increase in ACTH, in some cases with the detection of 21-hydroxylase antibody (21-OH Ab) and adrenal cortex antibody (ACA) (7, 8). Imaging often indicates bilateral enlargement of the adrenal glands with clear margins. Secondary adrenal insufficiency presents with a random or early-morning decrease in cortisol without a substantial increase or even a decrease in ACTH and may be combined with a decrease in other endocrine hormones of the pituitary gland (e.g. TSH, LH, or FSH) (9). The reduced blood cortisol level and significantly elevated ACTH were consistent with primary adrenal insufficiency (Addison’s disease). Moreover, although adrenal crisis was diagnosed at the same time as diabetes mellitus, it may have appeared earlier and presented only as mild hyponatremia or it may have appeared after the major stress of diabetic ketoacidosis.

Various difficulties were encountered during the treatment of this patient. (1) The lack of glucocorticoids reduced the ability to regulate blood glucose; accordingly, the patient was more prone to large fluctuations in blood glucose and was at higher risk of hypoglycaemia than that of other patients with type 1 diabetes. (2) Insufficient hydrocortisone supplementation induces adrenal crisis, whereas excessive supplementation is detrimental to glycaemic control. Therefore, every effort needed be made to maintain a balance between the doses of supplemental glucocorticoids and insulin.

**Figure 4:**
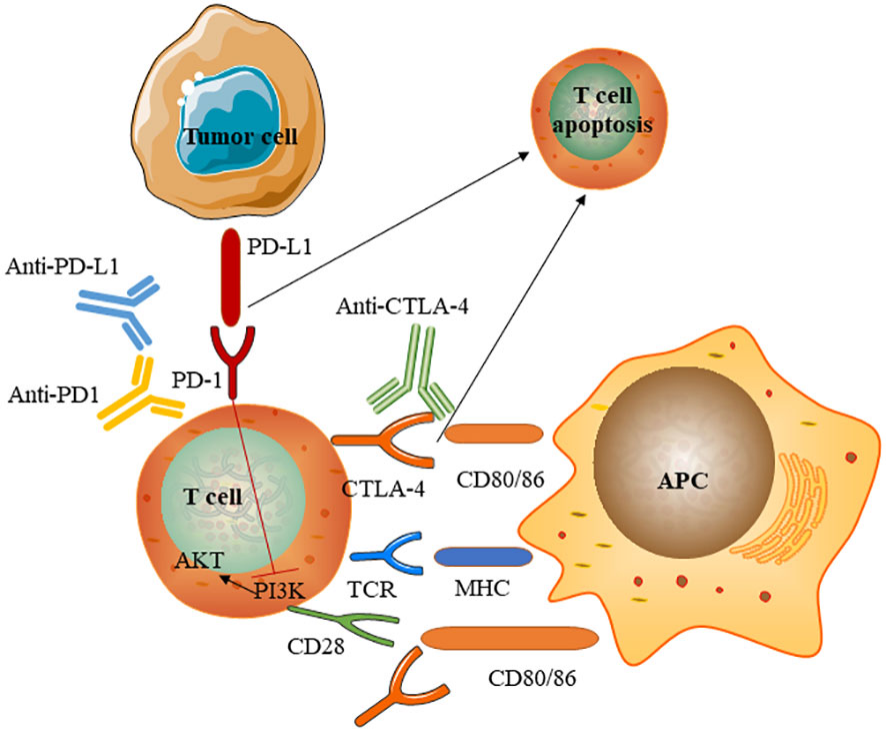
Mechanism of immune checkpoints and immune checkpoint inhibitors. Immune checkpoint inhibitors are monoclonal antibodies that target the CTLA-4 and PD-1 receptors and the PD-1 ligand PD- L1.T cell activation requires two signals: first, antigen recognition by the T cell receptor (TCR) following antigen presentation by major histocompatibility complex (MHC) class II molecules on the surface of antigen- presenting cells(APC); and, second, signal modulation by CD80 or CD86 binding to the CD28 receptor. CTLA-4 expressed on conventional T-cells interacts with its ligands CD80 and CD86 to initiate inhibitory signalling, enhance adhesion, and compete with CD28 to block interaction between CD28 and CD80/CD86.CTLA-4 inhibitors block CTLA-4 binding to CD80 or CD86 to promote T-cell activation PD-1 is a surface receptor that is expressed by T cells and promotes apoptosis of antigen- specific T cells and reduces apoptosis of regulatory T cells through its interaction with its ligand, PD- L1, which is expressed by tumour cells. Engagement of PD-1 with PD-L1 induces an intracellular inhibitory pathway to inhibit costimulatory CD28-activated PI3K pathways, leading to T-cell exhaustion. PD-1 and PD- L1 inhibitors block the PD-1–PD- L1 interaction, facilitating T cell activation.

Immune checkpoints are small molecules present on the cell surface of T-lymphocytes. They play critical roles in maintaining immune homeostasis and self-tolerance and modulating the duration and amplitude of physiological immune responses. Some immune checkpoints, such asCTLA-4 and PD-1, mediate inhibitory signals to blunt T-cell activity. ICPis serve to induce an anti-tumour immune response by blocking immune checkpoints. Normally, immune checkpoints downregulate T cell responses and act to protect the body from possibly damaging immune responses, such as autoimmune disease. However, tumours can evade the immune system through the activation of immune checkpoints and inhibition of the T cell response. Although the exact mechanism by which ICPis induce apoptosis remains unclear, We briefly summarise the mechanism of action of immune checkpoints and immune checkpoint inhibitors (**Figure 4**) (10, 11). The mechanisms underlying immune-related adverse events owing to ICPis depend on the type of ICPis therapy used (anti-PD1 or anti-PD-L1 versus anti-CTLA-4). anti-CTLA-4 can induce several cellular alterations, such as T cell activation and proliferation, impaired CD4+CD25+ regulatory T cell (Treg cell) survival and increased counts of type 17 T helper cells, in addition to the induction of cross-reactivity between anti-tumour T cells and antigens on healthy cells and autoantibody production. PD-1 and PD- L1 inhibitors reduce Treg cell survival and increase cytokine production (12).

A meta-analysis has revealed that endocrine disorders are among the most common irAEs in ICPis therapy (13). In particular, anti-CTLA-4 therapy was associated with pituitary inflammation, anti-PD1 therapy was associated with thyroid dysfunction, and the combination of anti-CTLA-4 and anti-PD1 therapy was associated with the highest incidence of ICPis-associated endocrine disorders. ICPis-associated endocrine disorders typically occur within 12 weeks after the start of ICPis treatment (13); however, they can occur months to years after ICPis are started. Some ICPis-related endocrine disorders may resolve spontaneously, but adrenal insufficiency and primary hypothyroidism are typically persistent (13). Similarly, in another meta-analysis, the most common endocrine disorders were hypothyroidism and hyperthyroidism, followed by hyperglycaemia, thyroiditis, and adrenal insufficiency. The risk ratios for adverse events were RR = 26.09% for adrenocortical insufficiency, RR = 26.67% for pituitary inflammation, and RR = 26.92% for hypopituitarism (14).

To review all published cases of APS-2 triggered by anti-PD1, PubMed was searched using the terms “autoimmune polyendocrine syndrome type 2” and “immune checkpoint inhibitors,” revealing 34 relevant case reports since 2000 (**Table 2**). All 34 patients were treated with anti-PD1, of which five were treated with anti-PD1 in conjunction with anti-CTLA-4. The largest proportion of patients were in the United States (9/34, 26.4%), with other cases reported in various countries, including France, Australia, the United Kingdom, and Ireland. The median age at diagnosis of APS-2 was 61 years. Of the 34 patients, 15.7% (5/34) had a history of autoimmune thyroid disease. The tumour types were primarily melanoma (20/34, 58.8%) and lung cancer (7/34, 20.6%), with cases of renal cell carcinoma and cervical cancer. APS-2 includes type 1 diabetes, autoimmune thyroid disease, and Addison’s disease. Among them, 88.2% (30/34) developed autoimmune thyroid disease, 85.3% (29/34) developed type 1 diabetes, 35.3% (12/34) developed Addison’s disease, and related autoantibodies were detected in 58.8% (20/34) of cases.

**Table 2:** Summary of case reports of APS-2 induced by anti-PD1.

First author	Year	Age (y)	Gender	Country	Tumour type	PD-1 inhibitors	Time^a^ (weeks)	First onset disease	Conditions	Auto-Ab	HLA type	Response
Hakami (15)	2019	52	Male	Ireland	melanoma	pembrolizumab	21	hypothyroidism	Primary hypothyroidism T1DM	(-)	NR	CR
Sakurai (16)	2018	68	Female	Japan	RCC	nivolumab	2	Painless thyroiditis	Thyroiditis^*^ (hypothyroidism) T1DM (fulminant T1DM)	TPOAb(_+_) TgAb(+)	DRB1*09: 01 DQB1*03: 03	CR
Gauci (17)	2017	73	Male	France	melanoma	nivolumab	6	DKA	Graves’ disease^*^ (hyperthyroidism) T1DM (fulminant T1DM)	GADA(+) IA2A(+) ZnT8A(+)	NR	CR
Paepegaey (8)	2017	55	Female	France	melanoma	pembrolizumab	12	thyroiditis	Thyroiditis (primary hypothyroidism) autoimmune adrenalitis (adrenal crisis)	anti-21-OH(+) ACA(+)	NR	PD
Li (18)	2017	63	Male	USA	NSCLC	nivolumab	4	DKA	T1DM Thyroiditis (Primary hypothyroidism)	GADA(+) TPOAb(+)	NR	PD
Alhusseini (19)	2017	65	Male	USA	NSCLC	pembrolizumab + ipilimumab	3	DKA	T1DM Thyroiditis (hyperthyroidism/hypothyroidism)	GADA(+) isletAb(+) IAA(+) TPOAb(+)	NR	PR
Lowe (20)	2016	54	Male	USA	melanoma	nivolumab + ipilimumab	2	Autoimmune thyroiditis	autoimmune thyroiditis (hyperthyroidism/hypothyroidism) T1DM (fulminant T1DM) hypophysitis (adrenal crisis)	GADA(+) TRAb(+) microsomalAb(+)	DQB1*0602	CR
Kong (21)	2016	68	Male	Korea	NSCLC	pembrolizumab	21	DKA	T1DM (fulminant T1DM) Subclinical hyperthyroidism	(-)	DRB1*09: 01 DQB1*03: 03	PR
Hansen (22)	2016	58	Male	USA	melanoma	pembrolizumab	NR	hypothyroidism	hypothyroidism T1DM	GADA(+)	NR	CR
Mellati (23)	2015	66	Female	USA	SCC jaw	PD-1 inhibitor	7	DKA	T1DM hypothyroidism	TPOAg(+) GADA(+)	DR3-DQ2 DR4-DQ8	NR
Hughes (24)	2015	55	Female	USA	melanoma	nivolumab + ipilimumab	20	NR	autoimmune thyroid disease^*^(hypothyroidism) T1DM	(-)	A2.1+ DR4+	NR
Hughes (24)	2015	64	Female	USA	melanoma	pembrolizumab	< 4	NR	autoimmune thyroid disease^*^(hypothyroidism) T1DM	(-)	DR4+	NR
Gunjur (4)	2019	78	Female	Italy	melanoma	pembrolizumab	3	DKA	T1DM (fulminant T1DM) AD (acute adrenal crisis) Primary hypothyroidism	GADA(+) IA-2A(+)	DRB1*04.16 DRB1*02.05 DRB1*01.03	CR
Hofmann (25)	2016	70	Female	Germany	melanoma	nivolumab	NR	NR	T1DM hyperthyroidism	(-)	NR	CR
Gaudy (26)	2015	44	Female	NR	melanoma	pembrolizumab	NR	DKA	T1DM (fulminant T1DM) autoimmune thyroiditis^*^	(-)	NR	CR
Scott (27)	2018	58	Male	Australia	melanoma	pembrolizumab + ipilimumab	2	subclinical hyperthyroidism	T1DM subclinical hyperthyroidism	(-)	NR	NR
Kurihara (28)	2020	48	Male	Japan	Parotid gland adenocarcinoma	nivolumab	16	thyrotoxicosis	T1DM Graves’ disease	TRAb(+)	DRB1*04:05	PD
Filette (29)	2019	61	Male	Belgium	NSCLC	pembrolizumab	8	DKA	T1DM Thyroiditis (Subclinical hyperthyroidism)	GADA(+)	DRB1*04 DQA1*03:01 DQB1*03:02	NR
Hong (30)	2020	76	Male	Korea	Lung cancer	pembrolizumab	11	DKA	T1DM Addison’s disease	(-)	NR	PR
Galligan (31)	2018	82	Male	Australia	SCC (oropharynx)	pembrolizumab	9	hyperglycaemia with ketosis	T1DM hypothyroidism	(-)	DRB1*04 (DR4) DQB1*03:02 (DQ8)	SD
Galligan (31)	2018	23	Male	Australia	melanoma	pembrolizumab	8	DKA	T1DM Thyroiditis (hypothyroidism)	GADA(+) TPOAg(+) IAA (+)	DRB1*03 (DR3), DRB1*04 (DR4), DQB1*03:02 (DQ8)	PR
Marchand (32)	2019	83	Male	Belgium	melanoma	pembrolizumab	NR	NR	T1DM Hashimoto’s disease	TPOAg(+)	DRB1*01:01 DQA1*01 DQB1*05:01/ DRB1*16:01 DQA1*01PD DQB1*05:02”	PR
Marchand (32)	2019	65	Male	Belgium	melanoma	pembrolizumab	NR	NR	T1DM (Fulminant T1DM) Hashimoto’s disease	TPOAg(+)	DRB1*04:01 DQA1*02 DQB1*02:02/ DRB1*07:01 DQA1*03 DQB1*03:01	PD
Hescot (33)	2018	33	Female	France	SCC (cervical)	pembrolizumab	6	autoimmune thyroiditis	AD (adrenal crisis) Autoimmune thyroiditis (hypothyroidism)	TPOAg(+) 21-OHAb(+)	NR	PR
Humayun (34)	2016	55	Male	UK	melanoma	Ipilimumab/ pembrolizumab	2	pan-hypopituitarism	hypophysitis (adrenal insufficiency) T1DM	–	NR	SD
Kuru (35)	2017	83	Female	USA	melanoma	Nivomulab	20	thyroiditis	autoimmune thyroiditis hypophysitis (acute adrenal crisis)	TPOAg(+)	NR	PR
Marchand (36)	2017	55	Male	NR	pulmonary pleomorphic carcinoma	Nivolumab	19	DKA	T1DM hypophysitis (adrenal insufficiency)	–	NR	PR
Sum (37)	2018	75	Male	USA	melanoma	Nivolumab+ ipilimumab	NR	T1DM	T1DM hypophysitis (adrenal insufficiency) subclinical hypothyroidism	GADA(+)	NR	CR
Tzouli (38)	2018	56	Female	UK	NSCLC	Nivolumab	NR	DKA	T1DM (fulminant T1DM) hypothyroidism	GADA(+)	NR	PR
Okahata (39)	2019	52	Female	Japan	Breast cancer	Nivolumab	28	adrenal insufficiency	secondary adrenal insufficiency T1DM (fulminant T1DM)	–	DRB1*14:05, 14:06	NR
Lupi (40)	2019	80	Male	Italy	melanoma	Nivolumab	6	thyrotoxicosis	autoimmune hypothyroidism hypophysitis (acute adrenal crisis)	TgAb(+) TPO(+)	NR	SD
Lupi (40)	2019	43	Female	Italy	melanoma	Pembrolizumab	4	thyrotoxicosis	Autoimmune hypothyroidism T1DM hypophysitis (acute adrenal crisis)	TPOAb(+) APA(+)	DQB1*02 DQB1*0602 DQA1*0102	SD
Machado (41)	2019	55	Male	Portugal	NSCLC	Nivolumab	14	thyroiditis	painless thyroiditis hypophysitis (secondary adrenal insufficiency)	(-)	NR	PR
Mayumi (42)	2021	59	Male	Japan	oral mucosal malignant melanoma	nivolumab	4	hypothyroidism	hypothyroidism secondary adrenal insufficiency T1DM	NR	NR	NR

auto-Ab, auto-antibody; IAA, insulin antibody; IA–2A, insulinoma-associated protein 2 antibody; GADA, glutamic acid decarboxylase antibody; ZnT8A, zinc transporter 8 antibodies; TPOAb, thyroid peroxidase antibody; TgAb, thyroglobulin antibody; TRAb, thyroid-stimulating hormone receptor antibody; 21-OHAb,21-hydroxylase antibody; ACA, adrenal cortex antibody; APA, anti-pituitary antibody; DKA, diabetes ketoacidosis; T1DM, type 1 diabetes mellitus; AD, Addison’s disease; APS-2, autoimmune polyendocrine syndrome type II; HLA, human leukocyte antigen; SCC jaw, sarcomatoid squamous cell carcinoma of the jaw; NSCLC, non-small-cell lung carcinoma; CR, complete response; PR, partial response; NR, not reported, PD, progressive disease; SD, stable disease

Time^a^ (weeks): weeks ever since the start of anti-PD1 therapy to onset of APS-2. *auto-immune condition preceded treatment with anti-PD1 axis therapy.

There is no specific differential diagnosis for patients with APS-2. Autoantibodies such as thyroid peroxidase antibodies in AITD, GADA in type 1 diabetes mellitus(T1DM), and 21-OH Ab in Addison’s disease contribute to the diagnosis and follow-up (43). The relationship between ICPis-related T1DM and islet autoantibodies remains unclear, and islet autoantibodies are present in about half of cases according to a review (44). Compared to islet autoantibody-negative individuals, islet autoantibody-positive individuals have a shorter onset and a higher rate of diabetic ketoacidosis (45). However, some patients treated with ICPis are antibody-positive but do not develop diabetes (46). A Japanese study also reported no correlation between thyroid autoantibodies and ICPis-related thyroid dysfunction (47). Therefore, the relationship between endocrine gland damage caused by ICPis and autoantibodies remains unclear, and threshold values for treatment and outcome prediction warrant further study.

The present case emphasises that immunosuppression-induced endocrine adverse reactions may involve multiple glands, especially the coexistence of life-threatening diabetic ketoacidosis and adrenal crisis, making early recognition, rapid diagnosis, and timely treatment especially important. In particular, the following points are noted. (1) When ICPis damage one gland, it is important to consider whether there is concomitant autoimmune damage to other glands. (2) It is recommended that all patients with hypothyroidism be evaluated for adrenal function prior to treatment. Special attention should be paid to clinical manifestations after treatment with levothyroxine, and the combination of adrenal insufficiency should be considered if fatigue worsens or if nausea, vomiting, or other discomfort develops. If both hypothyroidism and hypoadrenalism are present, levothyroxine alone may induce adrenal crisis. (3) Physicians should be aware of concomitant adrenal insufficiency in patients with T1DM when the dose of insulin required is lower than before, and blood glucose monitoring should be enhanced when glucocorticoids are given to patients with hypoadrenalism.

With the increased use of ICPis in clinical practice, several guidelines/consensus statements recommend the management of immune-related adverse events caused by ICPis (3, 48). Detailed medical history (especially the history of endocrine and autoimmune diseases), rational baseline screening (including adrenal function, thyroid function, and blood glucose tests), and regular follow-up (monitoring changes in endocrine indexes and the development of symptoms and signs) are needed to detect endocrine gland damage in a timely manner and avoid life-threatening diabetic ketoacidosis, adrenal crisis, thyroid crisis, and other serious events.

## Data availability statement

The original contributions presented in the study are included in the article/supplementary material. Further inquiries can be directed to the corresponding author.

## Ethics statement

Ethical approval was not required for the studies involving humans because the study did not identify data. The studies were conducted in accordance with the local legislation and institutional requirements. The human samples used in this study were acquired from a by-product of routine care or industry. Written informed consent to participate in this study was not required from the participants or the participants’ legal guardians/next of kin in accordance with the national legislation and the institutional requirements. Written informed consent was obtained from the individual(s) for the publication of any potentially identifiable images or data included in this article.

## Author contributions

QP: Writing – original draft. PL: Writing – review & editing.

## Glossary

**Table:**

APS-2	autoimmune polyendocrine syndrome type 2
ICPis	immune checkpoint inhibitors
irAEs	immune-related adverse events
anti-PD1	anti-programmed cell death protein 1
anti-PD-L1	anti-programmed cell death ligand 1
anti-CTLA-4	anti-cytotoxic T-lymphocyte antigen 4
T1DM	type 1 diabetes mellitus
TgAb	thyroglobulin antibody
ACTH	adrenocorticotrophic hormone
FT3	free triiodothyroxine
FT4	free thyroxine
TSH	thyroid stimulating hormone
TPOAb	thyroid peroxidase antibody
LH	luteinising hormone
FSH	follicle-stimulating hormone
GH	human growth hormone
GADA	glutamic acid decarboxylase antibody
IA2A	insulinoma-associated protein 2 antibody
ZnT8A	zinc transporter 8 antibody
ICA	islet cell antibody
IAA	insulin antibody
DKA	diabetes ketoacidosis
21-OH Ab	21-hydroxylase antibody
CA	adrenal cortex antibody
